# Prevalence and Factors Associated with Hydatidiform Mole among Patients Undergoing Uterine Evacuation at Mbarara Regional Referral Hospital

**DOI:** 10.1155/2018/9561413

**Published:** 2018-04-01

**Authors:** Olivier Mulisya, Drucilla J. Roberts, Elizabeth S. Sengupta, Elly Agaba, Damaris Laffita, Tusabe Tobias, Derrick Paul Mpiima, Lugobe Henry, Ssemujju Augustine, Masinda Abraham, Twizerimana Hillary, Julius Mugisha

**Affiliations:** ^1^University of Goma, Goma, Democratic Republic of the Congo; ^2^Department of Pathology, Massachusetts General Hospital, Boston, MA 02114, USA; ^3^Department of Pathology and Laboratory Medicine, Indiana University School of Medicine, Indianapolis, IN 46202, USA; ^4^Mbarara University of Science and Technology, Mbarara, Uganda; ^5^Makerere University, Kampala, Uganda

## Abstract

**Objective:**

We sought to determine the prevalence of and factors associated with hydatidiform molar gestations amongst patients undergoing uterine evacuation at Mbarara Regional Referral Hospital (MRRH), Mbarara, Uganda.

**Methods:**

This was a cross-sectional study carried out from November 2016 to February 2017. All patients admitted for uterine evacuation for nonviable pregnancy were included. The study registered 181 patients. Data were collected on sociodemographics, medical conditions, obstetrics, and gynecological factors. The evacuated tissue received a full gross and histopathologic examination. Cases of pathologically suspected complete hydatidiform mole were confirmed by p57 immunohistochemistry. Data were analyzed using STATA 13.

**Results:**

The prevalence of hydatidiform mole was 6.1% (11/181). All detected moles were complete hydatidiform moles, and there were no diagnosed partial hydatidiform moles. Clinical diagnosis of molar pregnancy was suspected in 13 patients, but only 69.2% (9/13) were confirmed as molar pregnancies histologically. Two cases were clinically unsuspected. Factors that had a significant relationship with complete hydatidiform mole included maternal age of 35 years and above (aOR 13.5; CI: 1.46–125.31; *p*=0.00), gestational age beyond the first trimester at the time of uterine evacuation (aOR 6.2; CI: 1.07–36.14; *p*=0.04), and history of previous abortion (aOR 4.3; CI: 1.00–18.57; *p*=0.05).

**Conclusion:**

The prevalence of complete hydatidiform mole was high at 6.1%. Associated risk factors included advanced maternal age (35 years and above), history of previous abortions, and gestational age beyond the first trimester at the time of evacuations.

**Recommendations:**

We recommend putting in place capacity to do routine histopathological examination of all products of conception especially those at high risk for a molar gestation either by clinical suspicion or by risk factors including advanced maternal age, advanced gestational age, and history of previous abortion because of high prevalence of complete mole.

## 1. Introduction

Hydatidiform moles (HMs) are forms of gestational trophoblastic disease (GTD) that involve villous formation. They are characterized histologically by aberrant changes within the placenta. Specifically, the chorionic villi in these placentas show varying degrees of trophoblastic proliferation and oedema of the villous stroma. Hydatidiform moles are categorized as either complete hydatidiform moles (CHMs) or partial hydatidiform moles (PHMs) based on biology and genetics [1].

Hydatidiform mole is the premalignant form of gestational trophoblastic neoplasia. It is of clinical and epidemiological interest because of its potential for significant consequences for women's health [2].

Of the two forms of molar disease, complete hydatidiform moles are more clinically important as they have high propensity for persistence (requiring clinical intervention) or progression to choriocarcinoma. Of complete moles, 15–20% will continue on to develop gestational trophoblastic neoplasia, whereas <5% of partial moles do. Complete hydatidiform moles are androgenic gestations, typically diploid but occasionally tetraploid. Partial hydatidiform moles are triploid conceptuses with the extra haploid set of chromosomes being paternally derived. Both types of moles are typically followed clinically for persistence by serum hCG levels and clinical symptoms (e.g., vaginal bleeding and persistent pregnancy symptoms) [3].

The incidence of molar pregnancy varies by geographical region. It is generally believed that the incidence is high in developing countries. The incidence is higher in women younger than 20 years and older than 40 years of age. It is also higher in nulliparous women, in patients of low economic status, and in women whose diets are deficient in protein, folic acid, and carotene [4].

Besides age, history of failed pregnancy increases the incidence of GTD. For example, elective abortion and miscarriage are connected with increasing risk of molar pregnancy [1].

Molar pregnancies are one etiology for pregnancy failure. The gold standard for a molar diagnosis is by histopathologic examination of the products of conception. The practice of routine histopathologic evaluation of tissue obtained at the time of abortion has been the subject of debate because some authors think that it is not necessary as the clinical significance of findings is low with low incidence of HM [5].

The usual management of abortions is not sufficient to detect all molar pregnancies because tissue is not routinely submitted for histological examination especially in resource-limited regions [6].

By routine histopathologic assessment of products of first-trimester spontaneous abortions, important pathologies such as molar pregnancy and placental trophoblastic neoplasia can be diagnosed [7].

The histopathologic diagnosis of molar pregnancies can be difficult and often requires expertise in perinatal pathology [8].

Ancillary studies such as genotyping and ploidy analyses are helpful but not universally available. Immunohistochemistry (IHC) offered in many pathology laboratories can be diagnostic for complete hydatidiform moles taking advantage of their androgenetic origin.

p57 is the gene product of the paternally imprinted, maternally expressed gene *CDKN1C*, a cyclin-dependent kinase inhibitor gene located on chromosome 11p15.5 [9]. It is expressed preferentially off the maternal allele and is therefore not expressed in complete hydatidiform molar villous stroma.

Our study sought to determine the incidence of HM in our population of women requiring uterine evacuation for failed pregnancies at a large referral hospital in Western Uganda. We diagnosed HM by full gross and histopathologic examination and confirmed the diagnosis with p57 IHC and review by an experienced perinatal pathologist (DJR).

## 2. Materials and Methods

This was a cross-sectional study carried out from November 2016 to February 2017 to determine the prevalence and clinical factors associated with hydatidiform mole among patients undergoing uterine evacuation at Mbarara Regional Referral Teaching Hospital.

**Figure 1:**
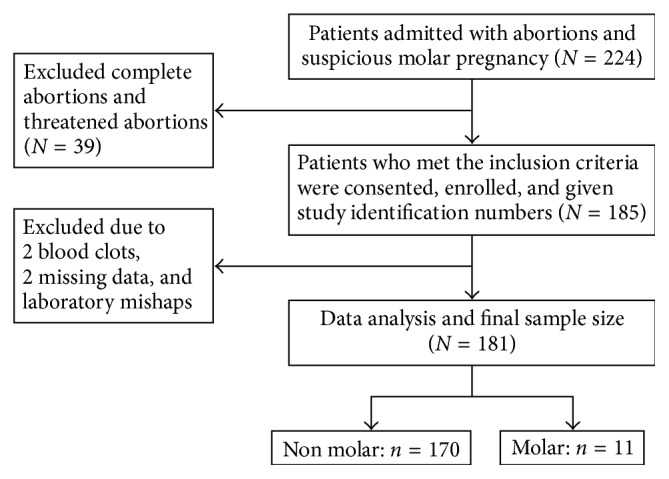
Flow diagram on recruitment.

Using the Kish formula, the minimum sample size was 175 [10]. Patients admitted with abortions both spontaneous and therapeutic including those suspicious for molar pregnancies by ultrasound were included in the study. See Figure 1 for recruitment strategy. All patients underwent ultrasonography before evacuation. The products of conception were kept in a container (500 ml jar) [11] with 10% buffered neutral formalin immediately after evacuation and taken to the MUST pathology laboratory for pathologic examination. Tissue processing was done using standard procedures: following fixation, the entire specimen was transferred to tissue cassettes and processed using an automatic Histokinette processor. Routine 5μ sections were stained with haematoxylin and eosin, and slides were evaluated by the MUST pathologists, and a subset were confirmed by a perinatal pathologist (DJR).

Immunohistochemical (IHC) p57 analysis was performed on all histopathologically diagnosed moles at the pathology laboratory at Massachusetts General Hospital in the USA. The IHC procedure employed a mouse monoclonal antibody (Leica Cat. # NCL-p57 Clone 25B2 diluted at 1:100), pretreated with EDTA pH 9.0 for 20 minutes and then stained on a BOND III autostainer. Slides were reviewed for immunoreactivity in the villous stroma using decidua as an internal positive control.

Data on sociodemographics, medical conditions, obstetrics, and gynecological factors were obtained by consultation with the referring clinicians. Patients were interviewed using a semistructured interviewer-administered questionnaire by the principal investigator and the research assistants. Questionnaire was filled in a private room in Gynaecological Ward after uterine evacuation. For age, parity, last normal menstrual period, history of previous abortion, type of previous abortion, contraceptive use, cigarette smoking, and tribe, we based on the participant's self-report. Univariate and multivariate analyses were computed using STATA 13.

## 3. Results

The mean age at presentation was 27.90 ± 7.1. Majority of the participants were married, Banyankole from the Mbarara district with a low socioeconomic status, and had attained a primary level of education. Majority of the participants were neither smokers nor alcohol consumers (Table 1).

**Figure 2:**
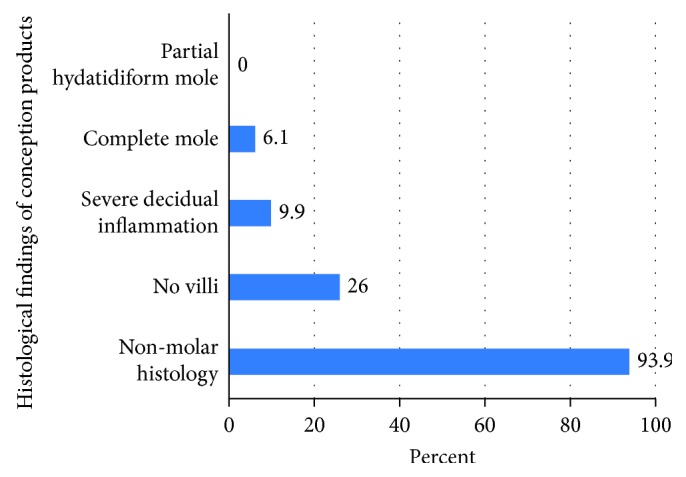
The histopathological findings.

By histopathology, the vast majority of the specimens were nonmolar (93.9%) (Figure 2), some with greater than usual inflammation (9.9%) and some without chorionic villi present (26%) but with gestational endometrium and blood clot only (presumed status after complete abortion).

**Figure 3:**
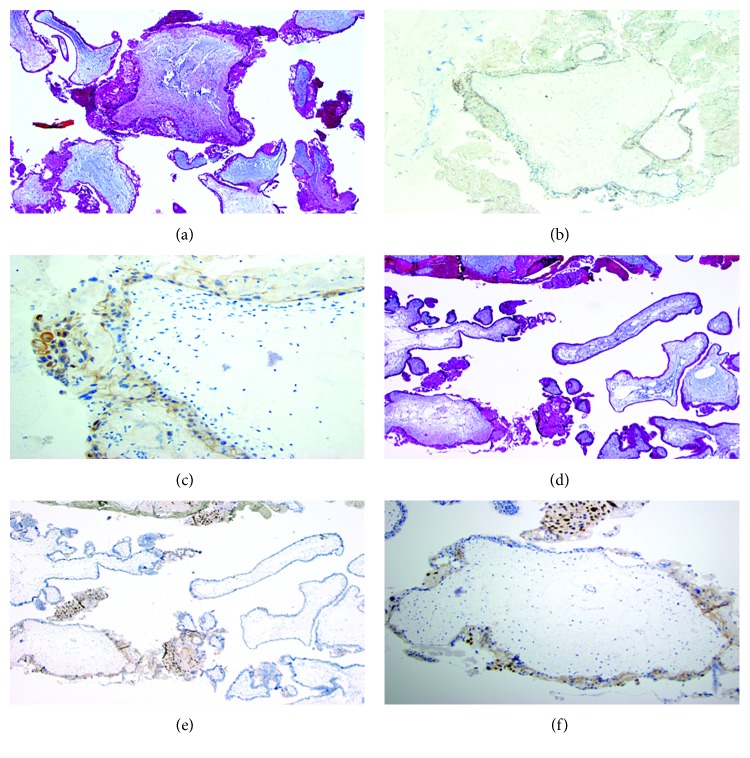
Complete hydatidiform mole slides. Histology and immunohistochemistry of two of the cases of complete hydatidiform mole. (a) Case MP115/17 H&E at 4x original showing enlarged villi with circumferential trophoblasts. Same case with p57 immunohistochemistry showing negative stromal and cytotrophoblast staining at 4x original (b) and 20x original (c). Note the syncytiotrophoblast stains focally positive in the nucleus and faintly in the cytoplasm. (d) H&E at 4x original of case MP1211/16; note the variety of villous sizes with cisternae and some with circumferential trophoblast hyperplasia. p57 immunohistochemistry at 4x original (e) and 10x original (f); note the lack of villous stromal reactivity but the presence of extravillous and villous syncytiotrophoblast staining.

The prevalence of hydatidiform mole was 6.1% (11/181), and all patients had complete mole confirmed by immunohistochemistry for p57 (Figure 3). Of the 13 patients with ultrasound scan diagnosis of molar pregnancy who underwent suction curettage, only 9 (69.2%) were confirmed to be complete moles, while 2 patients with clinically unsuspected molar pregnancy (by ultrasound scan) were identified after surgical evacuation for abortions (2/168; 1.2%).

Some numerical discrepancies are observed for contraception use as 50 (27.62%) study participants did not use any contraceptive method; for income, some women did not willingly want to give any information about their income; for the gestation age at evacuation, some women included in the study could not recall their last normal menstrual period.

As shown in Table 2, maternal age of 35 years and above, history of previous abortion, and gestational age beyond the first trimester at the time of uterine evacuation were found to be significantly and independently associated with complete mole. The odd of having complete mole is 13.5 times higher than in patients with maternal age of less than 25 years, 6.2 times higher in patients with gestational age beyond the first trimester, and 4.3 times higher in patients with history of previous abortion.

## 4. Discussion

In our study, the prevalence of hydatidiform mole was found at 6.1% among patients admitted for uterine evacuation. It is difficult to compare our prevalence with that in published reports due to the paucity of studies in sub-Saharan Africa with histopathologic validation of the diagnosis.

Our prevalence is lower than the rates of 12.8% in Tanzania reported in a cross-sectional study in a similar setting [12]. But in this study in Tanzania, there was no quality control by expert review or special studies. They reported 20/180 (11.1%) as partial mole and 3/180 (1.7%) as complete mole. The diagnosis of partial hydatidiform mole based solely on histopathology is difficult even for experienced pathologists [8]. Their report of 1.7% of complete moles is lower than our findings of 6.1%. We suggest that, in the study in Tanzania, many of the cases diagnosed as partial hydatidiform moles were in fact complete moles and many others likely nonmolar, but this would require reexamination of their histology to confirm. Our findings are in better agreement with other reports. For example, in Germany, Horn et al. found a similar prevalence, to ours, of 5.1% of HM, specifically complete hydatidiform mole confirmed with a molecular genotyping [13].

The preevacuation molar diagnosis (by ultrasound scan) in our study was observed in 13 patients, and 69.2% (9/13) were confirmed to be complete mole by histopathology, expert review, and immunohistochemistry diagnosis which has a higher suspicion rate compared to the study in the UK by Heath et al. where 2 (25%) were histologically confirmed molar pregnancy in 8 women diagnosed preoperatively by an ultrasound examination [14]. However, 2 patients with unsuspected molar diagnosis (2/168, 1.2%) were found to be complete mole in our study which follows between 1.16 and 12% as reported by Charry et al. [15].

Extremes of age have been a well-described risk factor for molar pregnancies [1]. We found that advanced maternal age was significantly associated with a diagnosis of hydatidiform mole. It is likely that the oocytes of the older women are more apt to unnatural fertilization [16]. Studies show a significant increase in risk in women with pregnancy above the age of 35 years and even further increase of 10-fold beyond the age of 40 years [17].

Previous history of abortion was reported in 44 (24.3%) participants of our study, and 7 (15.9%) of them were found to have complete mole. We found this history to be strongly associated with a diagnosis of hydatidiform mole, which is in accordance with others [1, 11, 18]. Hydatidiform mole was found to be more common in women with history of two or more abortions as well in the study in Ethiopia [18]. This could be due to the fact that many women do not know the nature of the previous abortion because histopathological examination is rarely done, yet hydatidiform mole may be one of the previous causes because history of hydatidiform mole has been established as a strong risk factor for subsequent hydatidiform mole [19].

However, our study cannot advance knowledge on the issue of the number of previous abortions and the ages at abortion of these patients.

In our study, the parity, socioeconomic status, blood group, and history of contraception use were not associated with hydatidiform mole.

There were no partial hydatidiform moles in our 181 cases. This may be because partial moles are more difficult to diagnose by morphology alone [20] but are likely due to our sample size. The published incidence of histopathologically and ploidy analysis verified partial hydatidiform mole is about 1 in 700 first- and second-trimester abortions [21], and rates in East Africa are not known. Although our perinatal pathologist did not review all 181 cases, she reviewed all the cases the MUST pathologist diagnosed as mole and 10% of other slides as part of quality control, and there was no discrepancy.

Our study is one of the largest studies in the literature of an Eastern African population. Our results highlight the utility/importance of histopathologic examination of products of conception. We suggest that all products of conception from high-risk patients (women of advance maternal age, those with histories of previous abortions, and those presenting beyond the first trimester) be examined pathologically to rule out a hydatidiform mole.

## 5. Conclusion

The prevalence of complete hydatidiform mole confirmed histopathologically by a perinatal pathologist and confirmed by p57 testing was high at 6.1%. The following factors were found to be independently associated with complete mole among patients evacuated at Mbarara Regional Referral Hospital: maternal age of 35 years and above, history of previous abortion, and gestational age beyond the first trimester at the time of evacuation. We recommend putting in place capacity to do routine histopathological examination of all products of conception because of high prevalence of complete mole. We recommend a cohort study aimed to determine risk factors of hydatidiform mole and to determine the outcome of patients with hydatidiform mole undergoing uterine evacuation at MRRH.

## Figures and Tables

**Table 1:** Sociodemographic and behavioral characteristics of patients.

Characteristic	*n*	*n* (%)
Age categories (years)	175	
<25		67 (38.3)
25–34		67 (38.3)
≥35		41 (23.4)
District	181	120 (66.3)
Mbarara		30 (16.6)
Isingiro		16 (8.8)
Kiruhura		15 (8.3)
Others		
Residence type	177	
Rural		93 (52.5)
Urban		84 (47.5)
Tribe	178	
Banyankole		157 (88.2)
Baganda		7 (3.9)
Bakiga		4 (2.2)
Others		10 (5.6)
Religion	177	
Catholic		51 (28.8)
Protestant		92 (52.0)
Moslem		19 (10.7)
Others		15 (8.5)
Highest level of education	179	
None		20 (11.2)
Primary		85 (47.5)
Secondary		55 (30.7)
Tertiary		19 (10.6)
Marital status	181	
Single		29 (16.0)
Married		152 (84.0)
Occupation	181	
Housewife		41 (22.6)
Business		45 (24.9)
Peasant		57 (31.5)
Student		9 (5.0)
Professional		29 (16.0)
Monthly income status	151	
<100k		89 (58.9)
>100k		62 (41.1)
Alcohol consumption	174	13 (7.5)
Cigarette smoking	180	4 (2.2)

**Table 2:** Univariate and multivariate logistic regression analyses for factors associated with hydatiform mole.

Variable	No CM, *n* (%)	CM, *n* (%)	cOR (95% CI)	*p* value	aOR (95% CI)	*p* value
Age categories (years)				**0.0013**		**0.0053**
<25	66 (98.5)	1 (1.5)	1.0		1.0	
25–34	65 (97.0)	2 (3.0)	2.0 (0.18–22.95)		1.5 (0.13–17.43)	
≥35	33 (80.5)	8 (19.5)	**16 (1.92–133.36)**		**13.5 (1.46–125.31)**	
Previous abortion						**0.051**
Yes	37 (84.1)	7 (15.9)	**6.3 (1.75–22.65)**	**0.0041**	**4.3 (1.00–18.57)**	
No	133 (97.1)	4 (2.9)	1.0		1.0	
Gestational age				**0.1718**		**0.042**
1st trimester	75 (97.4)	2 (2.6)	1.0		1.0	
2nd trimester	81 (91.0)	8 (8.9)	3.7 (0.76–18.00)		**6.2 (1.07–36.14)**	
Monthly income status				0.0243		
<100k	80 (89.9)	9 (10.1)	6.9 (0.85–55.63)			
>100k	61 (98.4)	1 (1.6)	1.0			
Parity				0.0916		
Nulliparous	49 (98.0)	1 (2.0)	1.0			
Primiparous	31 (96.9)	1 (3.1)	1.6 (0.09–26.20)			
Multiparous	70 (93.3)	5 (6.7)	3.5 (0.40–30.89)			
Grand multiparous	19 (82.6)	4 (17.4)	10.3 (1.08–98.31)			
Maternal blood group/rhesus status						
A+				NA		
Yes	31 (88.6)	4 (11.4)	2.6 (0.71–9.30)			
No	139 (95.2)	7 (4.8)	1.0			
A−				0.1888		
Yes	4 (100.0)	0 (0.0)	NA			
No	166 (93.8)	11 (6.2)	1.0			
B+				NA		
Yes	32 (88.9)	4 (11.1)	2.5 (0.68–8.93)			
No	138 (95.2)	7 (4.8)	1.0			
B−				NA		
Yes	2 (100.0)	0 (0.0)	NA			
No	168 (93.8)	11 (6.1)	1.0			
AB+				0.0867		
Yes	4 (100.0)	0 (0.0)	NA			
No	166 (93.8)	11 (6.2)	1.0			
O+				NA		
Yes	91 (96.8)	3 (3.2)	0.32 (0.83–1.27)			
No	79 (90.8)	8 (9.2)	1.0			
O−				0.1590		
Yes	3 (100.0)	0 (0.0)	NA			
No	167 (93.8)	11 (6.2)	1.0			
History of contraceptive use of oral pills				NA		
Yes	19 (86.4)	3 (13.6)	2.98 (0.73–12.21)			
No	151 (94.9)	8 (5.0)	1.0			
Progesterone only pills				0.8406		
Yes	6 (100.0)	0 (0.0)	NA			
No	164 (93.7)	11 (6.3)	1.0			
Injectaplan				0.6218		
Yes	72 (93.5)	5 (6.5)	1.1 (0.33–3.86)			
No	98 (94.2)	6 (5.8)	1.0			
Norplant				NA		
Yes	24 (96.0)	1 (4.0)	0.6 (0.74–4.97)			
No	146 (93.6)	10 (6.4).	1.0			
IUD				NA		
Yes	1 (100.0)	0 (0.0)	NA			
No	169 (93)	11 (6.1)				

CM, complete mole; cOR, crude odds ratio; CI, confidence interval; aOR, adjusted odds.
